# Financial and social impact of supporting a haematological cancer survivor

**DOI:** 10.1111/j.1365-2354.2011.01302.x

**Published:** 2011-11-10

**Authors:** M CAREY, C PAUL, E CAMERON, M LYNAGH, A HALL, F TZELEPIS

**Affiliations:** Research Centre for Health Behaviour, Faculty of Health, The University of NewcastleCallaghan, New South Wales, Australia

**Keywords:** financial support, social support, caregiver, oncology, support person

## Abstract

Support persons of haematological cancer survivors may be faced with unique challenges due to the course of these diseases and the treatments required. This study aimed to examine the social and financial impacts associated with their role. Eight hundred adult survivors of haematological cancer within 3 years of diagnosis were invited via an Australian state population-based cancer registry to complete a survey. Survivors were mailed two questionnaire packages, one for themselves and one for their primary support person. Non-respondents were mailed reminders via the survivor after 3 weeks. One hundred and eighty-two support persons completed the questionnaire (85% response rate). Of these, 67 (46%) support persons reported having at least one personal expense and 91 (52%) experienced at least one financial impact. Male support persons and support persons of survivors in active treatment reported experiencing more personal expenses than other support persons. Older participants reported fewer financial consequences. A greater number of social impacts were reported by those born outside Australia, those who had to relocate for treatment and support persons of survivors in active treatment. Future research should focus on practical solutions to reducing these impacts on support persons.

## INTRODUCTION

### The role of cancer support persons

Cancer support persons have been defined as those who a cancer survivor identifies as a significant source of support through their cancer diagnosis, treatment and follow-up (Campbell *et al*. 2009). As such the role of a support person, while overlapping with that of a carer, may reflect cancer survivors' diverse views of the support that they need, want and are able to obtain. Support persons may play a crucial role in the provision of emotional support (Thomas *et al*. 2002), assisting with finding information and providing practical support such as transport to appointments and help with household chores (Thomas *et al*. 2002). In some cases, support persons may also assist with care giving tasks such as assistance with personal care at home or helping to manage symptoms at home (Thomas *et al*. 2002).

### Challenges of the support person role

While very little research has focused on the experience of support persons, it is likely that like carers of people with cancer they experience significant emotional, social and financial impacts associated with their role. The care giving role has been shown to adversely affect physical health, resulting in sleep disturbance, fatigue, loss of appetite and weight loss (Given *et al*. 2003; Stenberg *et al*. 2010). Carers of people with cancer also experience higher rates of depression and anxiety than those in the general population (Gaugler *et al*. 2009), and may report feelings of hopelessness, frustration and fears about the future (Osse *et al*. 2006; Stenberg *et al*. 2010). A diagnosis of cancer may have considerable social (Taylor 2008) and financial consequences (Given *et al*. 2003; Sherwood *et al*. 2008; Tsigaroppoulos *et al*. 2009) resulting from changes in roles within the family, conflict, difficulty balancing new roles, and social isolation due to the demands of the care giving role (Given *et al*. 2003). Caregivers have also reported lost hours of work and financial strain (Given *et al*. 2003; Sherwood *et al*. 2008). For example, over half of the users of the Leukaemia Foundation's accommodation services in Queensland were receiving some type of financial benefit payment from the government, indicative of financial strain (McGrath 1998). Half reported travelling over 500 km to access treatment, and experienced a range of incidental costs associated with relocation (McGrath 1998).

### Limited research on the financial and social impact of supporting someone with haematological cancer

Collectively haematological cancers account for approximately 7% of all cancer diagnoses world-wide (Ferlay *et al*. 2010). Prognosis varies depending on the type of haematological cancer, with some requiring intensive inpatient treatments (National Institute for Clinical Excellence 2003; Cancer Council Australia 2010). As such, the support persons of haematological cancer survivors may be faced with unique challenges due to the types of treatment and course of these diseases (Tomayo *et al*. 2010) which may adversely impact on the social wellbeing of support persons, and have consequences for the types of financial impact experienced. Despite this, few studies have examined the experiences of support persons of haematological cancer survivors (McGrath 1998; Tomayo *et al*. 2010; Molassiotis *et al*. 2011). The limited research that exists suggests that key concerns of carers may relate to the impact of relocating for treatment (McGrath 2000), and meeting practical and information needs (Molassiotis *et al*. 2010). By assessing the specific types of financial and social impacts experienced by this group, there is potential to identify services or other types of support to address these needs.

This study aimed to: (1) describe support persons' perceptions of the number and type of (a) personal expenses; (b) financial impacts; and (c) social impacts associated with this role; (2) explore support persons' views about strategies which may reduce the financial and social impact of the support role; and (3) identify factors associated with experiencing a greater number of personal expenses, social and financial consequences, and endorsing a greater number of solutions to reduce these impacts.

## METHODS

### Design

A cross-sectional sample of principal support persons of survivors of haematological cancer was recruited. Ethical approval was obtained for this study prior to commencement.

### Participants and procedure

Survivors were recruited via an Australian state population-based cancer registry. Survivors were eligible if they were aged between 18 and 80 years and diagnosed with an ICD-10 and ICD-0-3 M defined haematological cancer including leukaemias, lymphomas (Hodgkin & Non-Hodgkin) and myeloma within the last 3 years. Eight hundred potentially eligible survivors were approached by the cancer registry via a letter inviting them to participate in a cross-sectional survey. On behalf of the researchers the cancer registry sent all eligible survivors a questionnaire package for themselves along with a separate questionnaire package for their primary support person. The support person questionnaire package included: a questionnaire, invitation letter, information sheet and reply-paid envelope. The primary support person was defined as ‘someone who has helped them the most during their cancer journey’. Survivors who did not respond to the initial questionnaire after 3 weeks were mailed a reminder letter from the cancer registry including a second questionnaire package for them and their primary support person. Return of a survey was taken as voluntary consent to participate in the study. Results of the survivor survey are reported elsewhere.

### Support person questionnaires

The following demographic characteristics were collected from the participants: gender, date of birth, postcode, marital status, Aboriginal and Torres Strait Islander status, education, employment status, household details (i.e. number of persons and relationship to those within the household), ethnicity, relationship to survivor and questions relating to the health of the support person and the person to whom they provide support. Age and time since the survivor's diagnosis were calculated from the date when invitations to participate in the research were sent (20 August 2010). Rurality was calculated from the support person's given postcode based on the Accessibility/Remoteness Index of Australia plus (ARIA+) codes (Australian Bureau of Statistics 2003). For the purpose of this study rural location was defined as any postal area code falling within the ARIA+ category of outer regional, remote Australia and very remote Australia. Whereas urban location was defined as any postal area code falling within the ARIA+ classification of major cities of Australia and inner regional Australia (Australian Bureau of Statistics 2003).

#### Personal expenses

Support persons were asked to indicate whether they had experienced any of the following personal expenses as a consequence of their support person role over the past month: travel to see family doctor, cancer specialists or other health professionals; accommodation while at hospital or cancer appointment; parking while at hospital or cancer appointment; drugs or treatments; homecare; medical supplies; child care; care for another family member; gardening or housework. Respondents were instructed to endorse as many of these options as were applicable.

#### Financial and social impacts

Support persons were asked whether, since being a support person, cancer tests or treatments had resulted in any of the following: had to take time off work; had less income; had to leave work or close your business; had difficulty with bills or other payments; had trouble with day-to-day expenses; had reduced access to children; needed help to care for family; missed family events or children's activities; missed important social or religious activities; lost contact with friends; used up your savings; had to borrow money. Respondents were asked to endorse as many options as were applicable to their circumstances.

#### Strategies to reduce the financial and social impact of being a support person

Seven potential strategies to reduce the financial and/or social impact of the support person role were presented. Such strategies included, but were not limited to ‘free parking at tests or treatments’, ‘appointments on weekends’, ‘direct financial assistance’ (for the full list of potential strategies see Fig. 2). Respondents were asked to indicate which of the strategies, if any, would assist with this aim. More than one strategy could be endorsed.

### Statistical analyses

Responses which were blank or incomprehensible were treated as missing data. For each of the four questions (personal expenses, financial impacts, social costs and solutions) the number of answers endorsed by the participant was counted and entered into an analysis separately as the dependent variable. Preliminary univariate analysis (Kruskall–Wallis non-parametric tests and non-parametric correlations for continuous variables) was conducted with the variables: age, gender, rurality, education, employment, birth country, if relocated for treatment, how many people they live with, if they live with someone under 18 years and the relationship, cancer diagnosis, time since diagnosis and treatment stage of the survivor. Those variables with a *P*-value less than 0.25 in the univariate analysis were included in negative binomial regressions to determine which variables accounted for the number of options endorsed. A backwards stepwise procedure which removed variables with a *P*-value less than 0.1 from the model in a stepwise fashion, was used.

## RESULTS

Of the 800 potentially eligible survivors, 68 were subsequently excluded as ineligible because they were non-contactable (56), had died (8), or were incorrectly diagnosed with a haematological malignancy (4). Of the 732 eligible survivors, 268 returned a survey, giving a response rate of 37%. A total of 182 support person surveys were received leaving 31 cases where the survivor indicated that they gave the survey to someone but it was not returned. The estimated support person survey response rate is therefore 85%. It is possible, that some additional non-responding survivors had given their support person a copy of the survey to complete making it difficult to establish the number of support persons who received a survey. The response rate may therefore, be an overestimate. Support persons were predominantly female, married, lived in an urban environment and were employed either full or part time (Table 1). In total, 91% of support persons lived with the cancer survivor they supported.

**Table 1:** Demographic characteristics of support persons

Variable	*n*= 182
Age (*n*= 180)	Mean = 57.9, SD = 13.0
Relationship to survivor (*n*= 181)	
Spouse/partner	82%
Relative	17%
Other	1.1%
Female (*n*= 182)	71%
Aboriginal and Torres Strait Islander (*n*= 181)	1.1%
Urban location (*n*= 181)	80%
Born in Australia (*n*= 182)	68%
Married or living with partner(*n*= 182)	93%
Education (*n*= 181)	
Primary	4.4%
Secondary	46%
Trade or vocational training	20%
University degree	29%
Employment status (*n*= 179)	
Full time	27%
Part-time	24%
Retired	33%
Do not do paid work	11%
Unable to work as caring for someone with cancer	3.3%
Residence (*n*= 182)	
Lives with family member	99.5%
Lives on own	0.5%
Lives with under 18 years old	17%
Survivor's age (*n*= 161)	Mean = 59.2, SD = 14.2
Time since survivor's diagnosis (months) (*n*= 160)	Mean = 18.4, SD = 10.2
Survivor in active treatment (*n*= 177)	11%
Survivor diagnosis (*n*= 179)	
Leukaemia	25%
Myeloma	16%
Non-Hodgkin lymphoma	51%
Other lymphoma	7%

### Financial impact of the support person role

**Figure 1:**
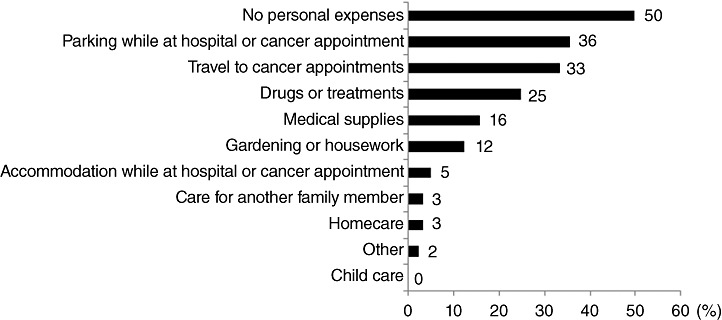
Percentage of support persons who have had personal expenses related to supporting someone with cancer by expense type (*n*= 173).

Half of the support persons reported having at least one of the personal expenses listed (mean number of expenses = 1.36, SD = 1.69, range = 7) (Fig. 1). A substantial proportion of participants reported that their role as a support person had a significant impact on their capacity to participate in paid work and earn an income. Ninety one (52%) of the support persons reported experiencing at least one of the financial impacts listed (mean number of impacts = 1.25, SD = 1.59, range = 6). Specifically, 70 (40%) participants reported that they had taken time off work, 50 (29%) reported having less income as a result of their role as a support person and 15 (8.6%) reported having to leave work or close their business. Support persons also indicated that their role had negative consequences for their personal finances. Thirty-four (19%) reported that they had used up their savings, 25 (14%) reported having difficulties with bills or other payments, 16 (9.1%) had trouble with day-to-day expenses and eight (4.8%) had to borrow money as a consequence of cancer-related expenses.

### Social impact of the support person role

Support persons reported that their role had adverse effects on their family and social life. Forty (23%) reported missing family events or children's activities, 28 (16%) missed social or religious activities, 28 (16%) lost contact with friends, 13 (7.4%) needed help to care for their family, and 12 (6.9%) had reduced access to their children as a consequence of the role as a support person. Sixty-six (38%) participants reported no financial or social impacts due to their support role.

### Strategies to reduce the social and financial impact of being a support person

**Figure 2:**
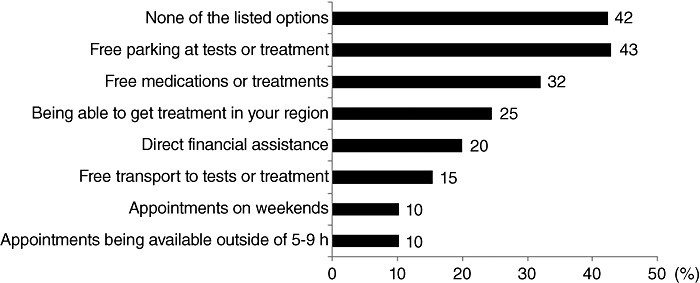
Strategies perceived to help reduce the financial and social impact of supporting someone with cancer (*n*= 176).

Of the solutions proposed 58% thought that at least one would help reduce the effect of cancer treatment on their finances, work, home or social activities (mean = 1.55, SD = 1.74, range = 7), with ‘free parking at tests or treatments centres’ the most highly endorsed strategy. The proportion endorsing each potential strategy as useful is shown in Figure 2.

Negative Binomial Regression revealed two variables associated with the number of personal expenses experienced by the support person (Table 2). Male support persons and support persons of survivors in active treatment reported experiencing more types of personal expenses than did other support persons. Fewer financial consequences were experienced by older participants, men and those who had retired compared to those in full- or part-time employment. A greater number of social impacts were reported by those born outside Australia, those who had to relocate for treatment and support persons of survivors in active treatment. Relocating for treatment and younger age were significantly associated with endorsing a greater number of strategies as being helpful to reducing the financial and social impact of the support person role.

**Table 2:** Factors associated with having a greater number of financial and social impacts, and endorsing a greater number of solutions to these impacts

	*n*	Mean number of costs or solutions (SD)	*P*	Incident rate ratio	95% Confidence interval
Number of personal expenses (*n*= 157)					
Gender					
Male	47	1.81 (1.83)	0.031	1.65	1.05–2.59
Female*	110	1.06 (1.57)			
Survivor treatment stage					
Pretreatment	18	0.89 (1.71)	0.003	0.28	0.12–0.65
Active*	19	3.21 (1.75)			
Maintenance	32	1.28 (1.76)	0.014	0.42	0.21–0.84
Follow-ups	47	0.98 (1.38)	0.001	0.33	0.17–0.63
In remission	41	0.93 (1.31)	<0.001	0.29	0.15–0.56
Number of financial impacts (*n*= 156)					
Age	156		<0.001	0.960	0.94–0.98
Gender					
Male	47	0.62 (1.05)	0.049	0.58	0.332–0.997
Female*	109	1.34 (1.58)			
Employment					
Full time	38	1.45 (1.33)	0.023	2.30	1.12–4.70
Part time	39	1.64 (1.37)	0.021	2.23	1.13–4.41
Do not do paid work	25	1.48 (2.1)	0.09	1.92	0.90–4.06
Retired*	54	0.35 (0.93)			
Number of social impacts (*n*= 156)					
Birth country					
Australia*	105	0.59 (0.94)			
Other	51	0.88 (1.01)	0.001	1.99	1.30–3.04
Relocated for treatment					
Yes	25	1.48 (1.12)	<0.001	2.85	1.87–4.35
No*	131	0.53 (0.86)			
Survivor treatment stage					
Pretreatment	17	0.18 (0.73)	0.001	0.13	0.04–0.45
Active*	18	1.28 (1.23)			
Maintenance	32	0.84 (1.05)	0.032	0.53	0.3–0.95
Follow-ups	46	0.52 (0.78)	<0.001	0.32	0.17–0.58
In remission	43	0.7 (0.94)	0.033	0.52	0.28–0.95
Number of solutions (*n*= 156)					
Age	156		0.045	0.980	0.97–0.999
Relocated for treatment					
Yes	24	2.63 (2.02)	0.009	1.89	1.17–3.06
No*	132	1.32 (1.59)			

* Reference category.

## DISCUSSION

The majority (82%) of the survivor-defined support persons who participated in the present study were either the spouse or partner of the person with cancer. Previous studies have reported that the proportion of spousal/partner carers ranges from 43% (Tsigaroppoulos *et al*. 2009) to 80% (Tomayo *et al*. 2010). Reviews on carers of people with cancer suggest that while most are the partner of the person with cancer, other family members such as adult children, parents, siblings, and in a minority of cases friends take on this role (Pitceathly & Maguire 2003; Stenberg *et al*. 2010). Similarly, other research suggests that family and friends are the most significant source of support following treatment for a haematological cancer (McGrath 2000).

### Financial impact of supporting someone with cancer

Tsigaropoulos and colleagues reported that 51% of caregivers of people with advanced cancer experience financial hardship (Tsigaroppoulos *et al*. 2009). While the present study did not directly assess financial hardship for support persons, it is notable that half the participants reported some type of out-of-pocket expense related to their support role. The most commonly reported expenses related to parking (36%), transport (33%), drugs or treatments (25%) and medical supplies (16%). These findings concur with those in a literature review assessing the impact on family caregivers of elderly survivors with cancer, which highlighted that non-reimbursable costs related to transport, meals away from home and travel were major out-of-pocket expenses borne by families (Haley 2003).

Forty per cent of support persons reported taking time off work to fulfil their role as a support person, having less income (29%) and having to use up their savings (19%). A smaller number of participants reported difficulties paying bills (14%) or meeting day-to-day expenses (9.1%). Similarly, the review of Stenberg *et al*. identified difficulty in paying bills, lack of sick leave/vacation time, loss of income and loss of savings and giving up work among the financial and social impacts of providing care to people with cancer (Stenberg *et al*. 2010).

Unsurprisingly, those supporting someone in active treatment were likely to report a greater number of personal expenses than other support persons. This may reflect that many of the items related to expenses are likely to be treatment-related such as parking and travel to attend the hospital, and medication expenses. More financial impacts were experienced by those in full- or part-time employment compared to retirees. This is likely to be due to the need to take time off work or leave work to fulfil the support person role. While male support persons reported a greater number of expenses than did female support persons, women felt a larger financial impact. This contrasts with previous research which found no association between gender and financial impact among partners of cancer survivors at 2 months, 2 years and 5 years post-diagnosis (Kim *et al*. 2010). Further research is needed to confirm the present finding.

### Social impact of supporting someone with cancer

A substantial minority of support persons reported that they had missed family events (23%), social or religious events (16%), and had less contact with their friends (16%) due to the demands of their role. Relatively few reported that they had needed help taking care of their family (7.4%) or had had less contact with their children (6.9%). This may reflect the age of the support persons in the present study. With a mean age of 57.9 it is possible that few of the support persons had dependent children. Only 17% reported living with someone under 18 years old. Alternatively it may suggest that either the support person role does not commonly interfere with these activities or that the support persons do not often ask for assistance with these tasks. This interpretation is consistent with a qualitative study in which caregivers of myeloma survivors reported that they felt they had to cope by themselves with little external support (Molassiotis *et al*. 2011). Those born outside of Australia, those supporting a person receiving active treatment, and those who had to relocate reported a greater number of social impacts than other support persons. While other research has indicated significant social consequences associated with re-location (McGrath 2000), the experiences of migrant carers have received little attention. Future work could provide a more in-depth assessment of the experiences of this group.

### Strategies which may help reduce the social and financial impact on support persons

Free parking (43%), free medication (32%) and being able to access treatment in your region (25%) were the most commonly endorsed strategies for reducing the social and financial impact for support persons. These findings are in accordance with the most commonly identified out-of-pocket expenses by the current sample. It is notable however, that 42% of respondents indicated that none of the listed options would reduce the effect of these impacts. Of these, 51% had not identified having any of the costs listed in previous questions. It is not clear whether this is because these respondents did not perceive that they experienced difficulty to manage financial or social consequences or whether they did not perceive that any of the options presented would alleviate these effects.

### Implications of findings

It appears that practical assistance in the form of free parking, assistance with travel and/or options to be treated closer to home, together with subsidies for medication may assist in reducing the social and financial burden associated with the support person role. Most of the burden on the support person, in terms of personal expenses and social costs, occurs when the survivor is in the active stage of treatment. Targeting solutions to this stage may help alleviate these effects. Further those who have had to relocate, and who are born outside Australia may be particularly in need of assistance to deal with the demands of the support person role.

### Limitations

#### Response rate

While the response rate for support persons could not be accurately calculated, given the modest response rate for survivors (37%), it is possible that the sample may not be representative of all support persons. Similar difficulties in obtaining a representative sample have been reported by previous research (Smith *et al*. 2007; Campbell *et al*. 2009). For example, Campbell and colleagues (Campbell *et al*. 2009, 2010) used a similar method to recruit cancer survivors and their support persons through a Canadian cancer registry. While a higher proportion of survivor participants (88%) reported giving their support person the survey than in the current study (79%), the return rate for support persons was only 38% (Campbell *et al*. 2009).

## CONCLUSIONS

It appears that a substantial proportion of support persons experience personal expenses and financial and social consequences as a result of their support role. Support persons of those currently receiving treatment, those who have had to relocate for treatment and migrants may experience greater impact of their role than other support persons. Future research should focus on practical solutions to reducing these impacts on support persons, and exploring the specific concerns experienced by high risk subgroups such as those born outside of Australia.
